# A case of primary cutaneous diffuse large B-cell lymphoma leg type presenting as lower extremity edema and ulcers

**DOI:** 10.1016/j.jdcr.2024.03.029

**Published:** 2024-11-29

**Authors:** Jonathon D’Gama, Elie Saliba, Andrew C. Walls

**Affiliations:** a Harvard Medical School, Boston, Massachusetts; b Section of Dermatopathology, Department of Dermatology, Boston University School of Medicine, Boston, Massachusetts; c Department of Dermatology, Brigham and Women’s Hospital, Boston, Massachusetts

**Keywords:** leg type, leg wound, lymphoma, wounds

## Introduction

Primary cutaneous diffuse large B-cell lymphoma, leg type is a rare malignancy, originally described as a separate entity in a 2005 classification of cutaneous lymphomas.^1^ It most commonly occurs on the lower extremities in elderly women, accounts for 20% of primary cutaneous B-cell lymphomas and is the most aggressive subtype. Survival rates are 70% with optimal therapy, although >50% of patients experience disease relapse.^2^, ^3^, ^4^ Clinically, it typically presents with infiltrative papules, plaques, or nodules on the legs; ulceration, is less common, and can clinically mimic common vascular ulcers.^5^^,^^6^ Diagnosis relies on skin biopsy with histologic findings of reticular dermal perivascular lymphocytic infiltrates and confluent sheets of atypical lymphocytes (centroblasts and immunoblasts positive for CD20, CD79a, BCL-2, and MUM-1).^1^^,^^4^

## Case report

A 75-year-old man with a history of longstanding Crohn’s disease treated with adalimumab was transferred from an outside hospital with leg ulcerations, erythema, pain, and computed tomography (CT) imaging concerning for compartment syndrome. Five months before admission, the patient had experienced bilateral lower extremity swelling, which rapidly progressed into ulcers. He had been followed by a local wound care clinic and was treated with silver dressings, oil emulsion dressings, compression, and numerous courses of antibiotics without any improvement. Because of progression of ulcers with malodor and significantly worsening pain, he presented to a local emergency department and was started on vancomycin and ceftriaxone given concern for cellulitis. A CT was performed and demonstrated: “cutaneous thickening over the anterior aspect of the leg, extensive intramuscular edema of the anterior, lateral, and deep compartments of the lower portion of the leg with loss of the fat planes highly concerning for compartment syndrome.” The patient was transferred to our tertiary care hospital for further management.

**Fig 1:**
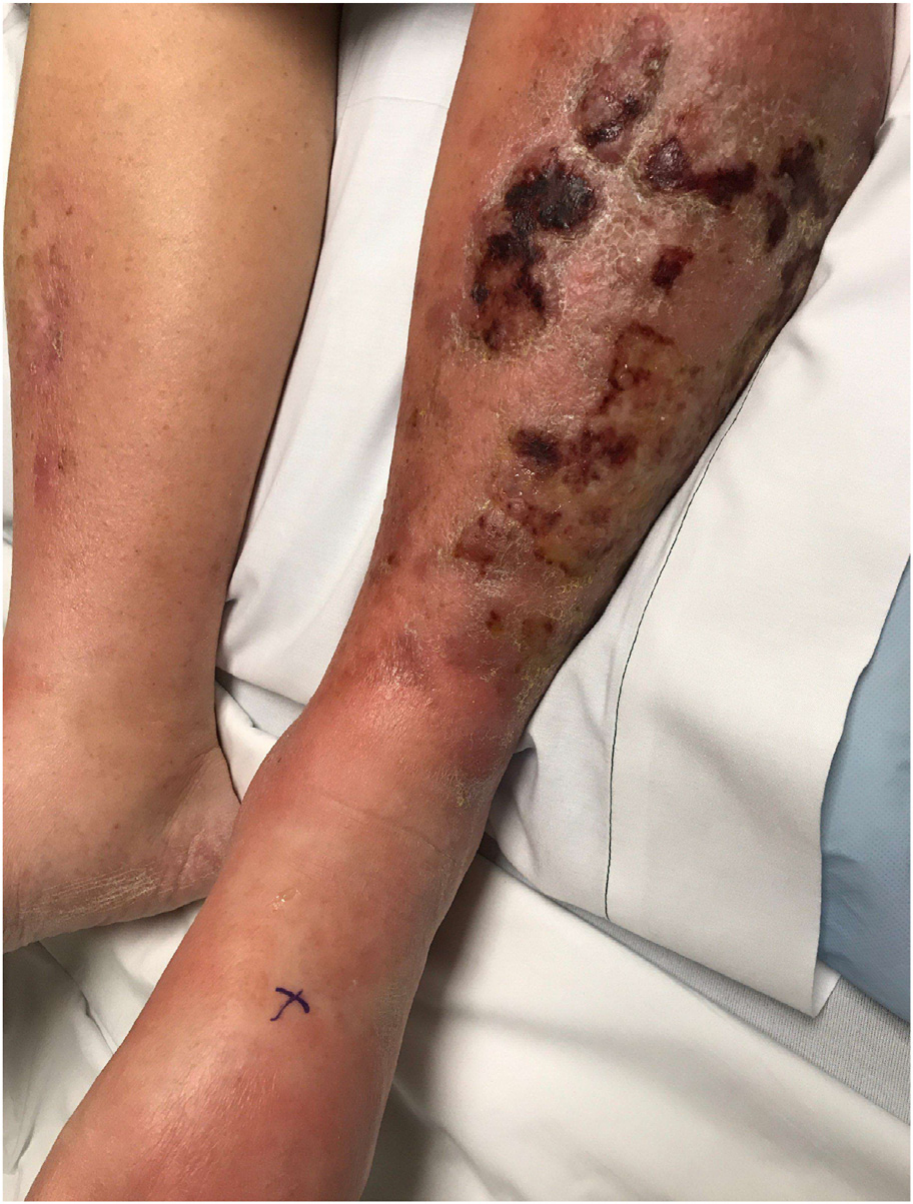
Lower portion of the left leg with scattered, multifocal superficial ulcerations, cobblestoning, and nodularity on a background of tender erythema and 3+ pitting edema to the thigh. Similar, although significantly diminished, findings were seen in the right leg.

Upon initial evaluation by the dermatology team, the patient had normal vital signs. He endorsed worsening pain to the point that he could no longer elevate his legs and had been unable to sleep. Physical examination demonstrated bilateral lower extremity pitting edema (3+) up to thighs. The left anterolateral shin had a hard, woody, nodular cobblestone texture superimposed upon diffuse erythema. Multiple ulcerations with overlying hemorrhagic and serous crusting were noted on the left, but not right side of the lower extremity (Fig 1). Laboratory evaluation was notable for elevated lactate dehydrogenase of 434 (normal range 135-225). Complete blood cell count revealed a normal white blood cell count but mild anemia (hematocrit = 26%; normal range: 36%-48%) and platelets (91; normal range 150-450 k/uL). Erythrocyte sedimentation rate and C-reactive protein were moderately elevated at 51 (normal 0-12 mm/h) and 15 (normal 0-10 mg/L), respectively.

Ultrasonography of the lower extremities demonstrated a nonvascular, poorly demarcated, hypoechoic mass present in the left calf, consistent with a fluid collection. CT angiography of the leg demonstrated: diffuse intramuscular edema with smaller muscular enlargement in the anterior, deep, and lateral compartments, and diffuse atherosclerotic disease throughout the lower extremities with severe calcification of both posterior tibial arteries.

The patient was seen in consultation by the Orthopedic, Vascular Surgery, and Infectious Disease services who believed his presentation was not consistent with compartment syndrome, surgically intervenable peripheral arterial disease or infectious myositis. Given the clinical appearance of lymphedema and pitting edema, the Dermatology service believed that wounds were most consistent with ulceration due to venous and lymphatic insufficiency. Recommendations for wound care, compression and close clinical follow-up were made.

**Fig 2:**
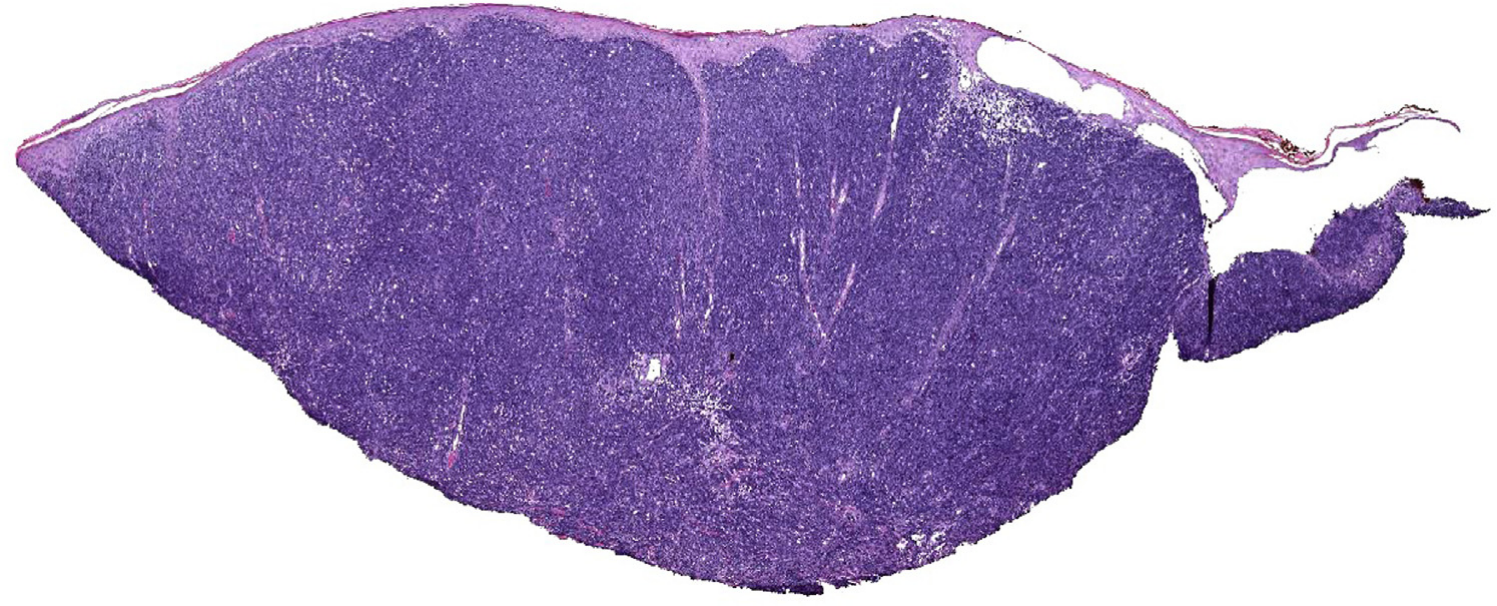
Primary cutaneous diffuse large B-cell lymphoma, leg type. Dense diffuse lymphoid infiltrates involving the entire dermis (Hematoxylin-eosin stain; original magnification: ×2).

**Fig 3:**
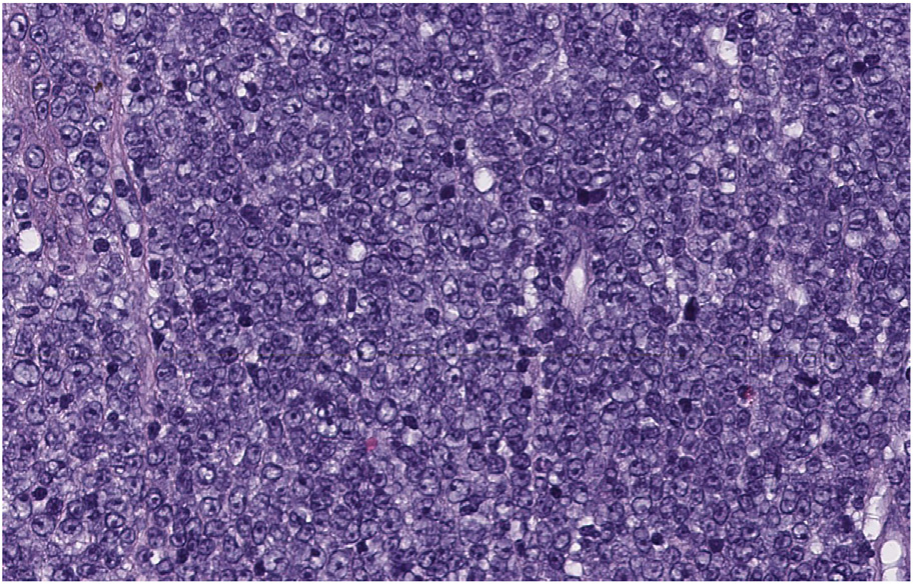
Round medium-to-large cytologically atypical and mitotically active immunoblasts and centroblasts (Hematoxylin-eosin stain; original magnification: ×40).

**Fig 4:**
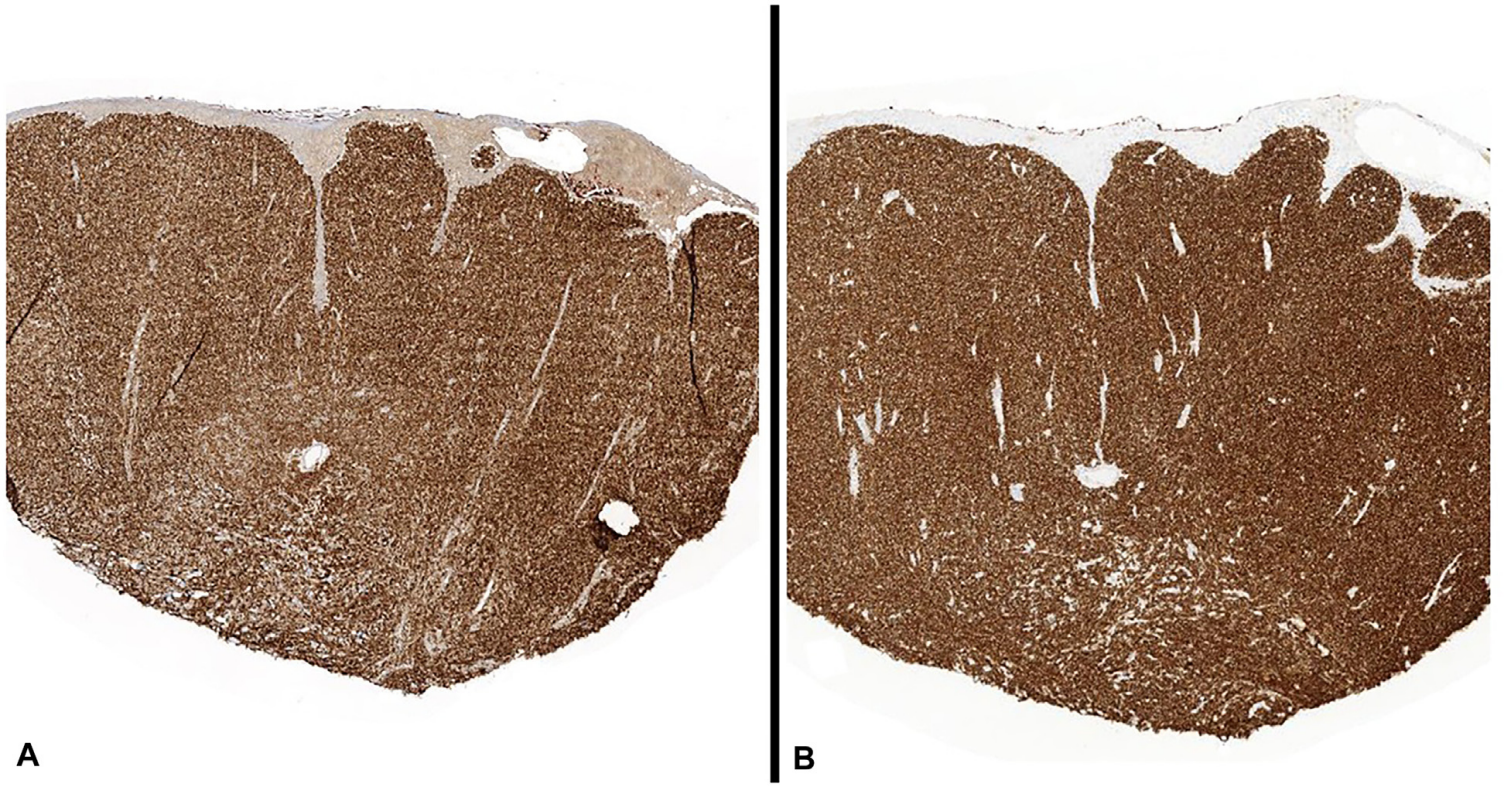
Immunohistochemistry demonstrating lesional cells are diffusely positive for (**A**) CD20 and (**B**) BCL-2.

Upon outpatient evaluation, the patient had endorsed no improvement in pain or appearance of the wounds. A diagnostic skin biopsy was performed demonstrating primary cutaneous diffuse large B-cell lymphoma, leg type (Figs 2 and 3) with supportive immunohistochemistry demonstrating CD20 and BCL-2 positivity(Fig 4), whereas negative for MUM-1, BCL-6, CD10, and CD138. At the time of diagnosis, initial positron emission tomography-CT staging scans were negative for distant disease. The patient underwent 3 cycles of rituximab, cyclophosmamide, hydroxydaunorubicin (doxorubicin), oncovin (vincristine), prednisone (R-CHOP) therapy. Repeat staging scans unfortunately demonstrated widespread, treatment-refractory metastasis. The patient withdrew from further care, passing away shortly thereafter.

## Discussion

Adalimumab (Humira) is a tumor necrosis factor-α monoclonal antibody used in a variety of rheumatologic and autoimmune disorders, including inflammatory bowel disorders. Although there were initial concerns for an association between lymphomas and tumor necrosis factor-α inhibitors, recent study of long-term adverse events of adalimumab in a large group of patients with Crohn’s disease found the lymphoma rate for patients on adalimumab was below the estimated background rate.^7^ However, there are case reports of adalimumab usage associated with development of cutaneous T-cell lymphoma and noncutaneous B-cell lymphoma but no reports of an association with any types of primary cutaneous B-cell lymphoma.^8^, ^9^, ^10^

Our patient had been misdiagnosed by multiple clinicians, including his local wound care clinic, emergency departments as well as multiple expert consultants after transfer to a tertiary medical center. The diagnosis was made difficult because of the rapid progression of disease, in that the tumors presented as ulcerations, there were clear-cut findings of significant venous and lymphatic insufficiency (due to tumor infiltration of regional nodes), as well as the fact that numerous radiological studies including, ultrasound, CT, and CT angiography did not suggest malignancy as a favored diagnosis. As such, skin biopsy was required for diagnosis and should be performed expeditiously for any wound without a clear etiology, or any wound not responding to appropriate standard treatment.^10^

## Conflicts of interest

None disclosed.
